# Comparative Outcomes of Super Thin Anterolateral Thigh Flaps Versus Radial Forearm Free Flaps in Head and Neck Reconstruction

**DOI:** 10.1002/micr.70278

**Published:** 2026-08-25

**Authors:** Kyung Jun Heo, Woo Yeol Baek, Won Jai Lee, Young Chul Suh

**Affiliations:** ^1^ Department of Plastic and Reconstructive Surgery Yonsei University College of Medicine, Severance Hospital Seoul Republic of Korea; ^2^ Institute for Human Tissue Restoration, Yonsei University College of Medicine Seoul Republic of Korea

**Keywords:** anterolateral thigh flap, head and neck reconstruction, microvascular surgery, salivary leakage, super thin flap

## Abstract

**Background:**

The anterolateral thigh (ALT) flap is a versatile reconstructive option for head and neck defects but may be excessively bulky in contour‐sensitive regions. Super thin ALT flaps (≤ 10 mm) have been introduced to improve pliability and contour; however, their comparative clinical advantages over established fasciocutaneous flaps remain unclear. This study aimed to evaluate the clinical outcomes of super thin ALT flaps and to compare them with radial forearm free flaps (RFFF) in head and neck reconstruction.

**Methods:**

A retrospective study was conducted on patients who underwent head and neck reconstruction with super thin ALT flaps between 2021 and 2023 at a single tertiary center. A comparative cohort of patients reconstructed with RFFF during the same period was also included. Super thin ALT flaps were elevated within the superficial fascial layer with a target thickness of ≤ 10 mm. Outcomes assessed included complication rates, fistula formation, reoperation, flap failure, and operative time. Postoperative drain amylase levels were analyzed as a surrogate marker for salivary leakage.

**Results:**

A total of 28 patients in the super thin ALT group and 44 in the RFFF group were included in the analysis. All ALT flaps survived without partial necrosis. There were no statistically significant differences between the groups in total complication rate (7.1% vs. 11.3%, *p* = 0.692), fistula formation (3.5% vs. 6.8%, *p* = 1.000), reoperation (7.1% vs. 11.3%, *p* = 0.692), or operative time (145 ± 34 min vs. 159 ± 42 min, *p* = 0.125). No flap failure occurred in the super thin ALT group, whereas three cases (6.8%) were observed in the RFFF group. The super thin ALT group demonstrated lower postoperative amylase levels, although the difference was not statistically significant.

**Conclusion:**

Super thin ALT flaps provide clinical outcomes comparable to those of RFFF while offering enhanced pliability and improved conformity to complex defects. These characteristics make them a reliable and versatile alternative for head and neck reconstruction, particularly in contour‐sensitive regions.

## 1 Introduction

Reconstruction of head and neck defects following oncologic resection or trauma often requires thin, pliable, and well‐vascularized soft tissue coverage. The anterolateral thigh (ALT) flap is widely used in such cases due to its consistent anatomy, long vascular pedicle, and versatility (Wei et al. 2002; Lonie et al. 2016). It provides a large skin paddle with acceptable donor site morbidity and is especially advantageous in centers with microsurgical expertise (Chana and Wei 2014). Previous studies have demonstrated the reliability of the ALT flap, particularly in achieving stable vascular outcomes and flexible flap design (Yu 2004; Chen and Tang 2003; Kimura and Satoh 1996).

However, one of the primary limitations of the traditional ALT flap is its inherent thickness, particularly in patients with elevated body mass index. This bulk can pose both functional and aesthetic challenges in regions requiring delicate contouring, such as the oral cavity, periorbital region, or floor of the mouth. Excess volume may impair articulation, mastication, and symmetry, and may hinder precise adaptation to complex three‐dimensional defects (Koshima et al. 2020).

In response to these limitations, the concept of the “super thin” ALT flap has been developed. By dissecting within the superficial fascial layer and preserving the subdermal plexus, surgeons can harvest a significantly thinner flap, thereby improving pliability and contour match without compromising vascularity (Hong et al. 2017; Bekara et al. 2015). This technique has gained increasing acceptance, and several studies have reported its safety and feasibility (Rajacic et al. 2002; Kimura et al. 2001; Hallock 2013).

However, despite these encouraging results, the comparative clinical advantage of super thin ALT flaps over established fasciocutaneous flaps—particularly the radial forearm free flap (RFFF)—remains insufficiently defined (Suh et al. 2021; He et al. 2012; Acosta et al. 2010). In particular, functional outcomes such as salivary leakage and fistula formation, which are critical in head and neck reconstruction, have not been adequately evaluated in comparative settings.

Therefore, the aim of this study was to evaluate the clinical outcomes and safety profile of super thin ALT flaps in head and neck reconstruction and to assess their potential advantages in contour‐sensitive regions. Additionally, a comparative analysis with RFFF was performed to better elucidate differences in postoperative outcomes, including fistula formation and salivary leakage.

## 2 Patients and Methods

### 2.1 Patients

We conducted a retrospective study involving patients who underwent head and neck reconstruction using super thin anterolateral thigh (ALT) flaps at Severance Hospital between January 2021 and December 2023. Inclusion criteria included patients who received a free ALT flap for primary or secondary reconstruction of oncologic or traumatic defects in the head and neck region. Patients with prior radiation to the donor site or without available intraoperative flap thickness measurements were excluded.

To provide a comparative analysis, a cohort of patients who underwent reconstruction using radial forearm free flaps (RFFF) during the same period was additionally identified. These cases were reviewed retrospectively using the same inclusion criteria where applicable.

### 2.2 Surgical Techniques

All ALT flaps in the study group were intraoperatively thinned to achieve a super thin profile, typically ≤ 10 mm, based on surgical judgment using sterile caliper measurements. Super thin elevation was performed within the superficial fascial layer, preserving the subdermal vascular plexus. No thinning was performed beyond the suprafascial layer to avoid perforator compromise. Flap thickness was measured intraoperatively at the central portion of the flap. All procedures were performed by a single reconstructive team experienced in ALT flap elevation and microvascular surgery.

For the RFFF group, flaps were harvested in a standard fasciocutaneous manner based on the radial artery and its venae comitantes. Flap thickness was not routinely modified. Microvascular anastomosis was performed using standard end‐to‐end techniques.

### 2.3 Assessments

Collected variables included patient demographics (age, sex, BMI), comorbidities, prior radiotherapy, defect location, flap dimensions, flap thickness, and pedicle length. Defects were categorized into tongue, buccal mucosa, gingiva, and oropharynx.

Primary outcome measures included postoperative fistula formation and overall complication rates. Fistula was defined as clinically evident salivary leakage requiring conservative or surgical management. Complications included wound infection, hematoma, seroma, and partial flap necrosis.

Secondary outcomes included complete flap survival, partial necrosis, unplanned re‐exploration, donor site complications, and requirement for revision surgery. Flap failure was defined as total loss of the flap requiring removal.

Drain amylase levels were collected as a surrogate marker for salivary leakage when available. In the super thin ALT group, amylase analysis was limited to patients with intraoral or pharyngeal defects (oral cavity, oropharynx, and hypopharynx), where salivary leakage could be assessed. Peak amylase levels and postoperative trends were analyzed.

### 2.4 Statistical Analysis

Descriptive statistics were used to summarize patient demographics and surgical characteristics. Continuous variables were compared using Welch's *t*‐test or Mann–Whitney *U* test as appropriate, and categorical variables were compared using Fisher's exact test. A *p*‐value < 0.05 was considered statistically significant.

## 3 Results

A total of 28 patients underwent head and neck reconstruction using super thin ALT flaps, and 44 patients underwent reconstruction using radial forearm free flaps (RFFF) during the study period. All super thin ALT flaps were elevated within the superficial fascial layer with preservation of the subdermal vascular plexus. The mean flap thickness in the ALT group was 8.8 mm (range, 7–10 mm).

Patient demographics and baseline clinical characteristics are summarized in Table 1. The two groups were comparable in terms of age, sex, and body mass index. Prior radiotherapy was more frequently observed in the super thin ALT group (35.7%) compared to the RFFF group (9.1%).

**TABLE 1:** Patient demographics and baseline clinical characteristics.

Variable	Super thin ALT (*n* = 28)	RFFF (*n* = 44)
Age (mean ± SD)	60.9 ± 10.1 years	57.3 ± 8.6 years
Sex (M/F)	18/10	31/12
BMI (mean ± SD)	24.3 ± 2.7 kg/m^2^	24.8 ± 2.3 kg/m^2^
Etiology of Defect (*n*, %)		
Oral cavity cancer	14 (50.0%)	32 (72.7%)
Oropharyngeal cancer	4 (14.3%)	7 (15.9%)
Hypopharyngeal cancer	3 (10.7%)	5 (11.3%)
Reconstructive revision	2 (7.1%)	0
Trauma	2 (7.1%)	0
Others	3 (10.7%)	0
Prior radiotherapy (*n*, %)	10 (35.7%)	4 (9.0%)
Prior chemotherapy (*n*, %)	6 (21.4%)	3 (6.8%)

Comparative surgical outcomes between the two groups are presented in Table 2. There were no statistically significant differences in total complication rates (7.1% vs. 11.3%, *p* = 0.692), major complication rates (3.6% vs. 11.3%, *p* = 0.387), or reoperation rates (7.1% vs. 11.3%, *p* = 0.692) between the super thin ALT and RFFF groups.

**TABLE 2:** Comparison of surgical outcomes between super thin ALT flap and radial forearm free flap.

Outcome	Super thin ALT (*n* = 28)	RFFF (*n* = 44)	*p*
Total complication	2 (7.1%)	5 (11.3%)	0.692
Major complication	1 (3.5%)	5 (11.3%)	0.387
Fistula rate	1 (3.5%)	3 (6.8%)	1.000
Reoperation	2 (7.1%)	5 (11.3%)	0.692
Flap failure	0	3 (6.8%)	0.266
Operative time	145 ± 34	159 ± 42	0.125

The incidence of postoperative fistula formation was low in both groups and did not differ significantly (3.5% vs. 6.8%, *p* = 1.000). Notably, no flap failure occurred in the super thin ALT group, whereas three cases (6.8%) of flap failure were observed in the RFFF group, although this difference was not statistically significant (*p* = 0.266).

The mean operative time was 145 ± 34 min in the super thin ALT group and 159 ± 42 min in the RFFF group, with no statistically significant difference between groups (*p* = 0.125).

**FIGURE 1:**
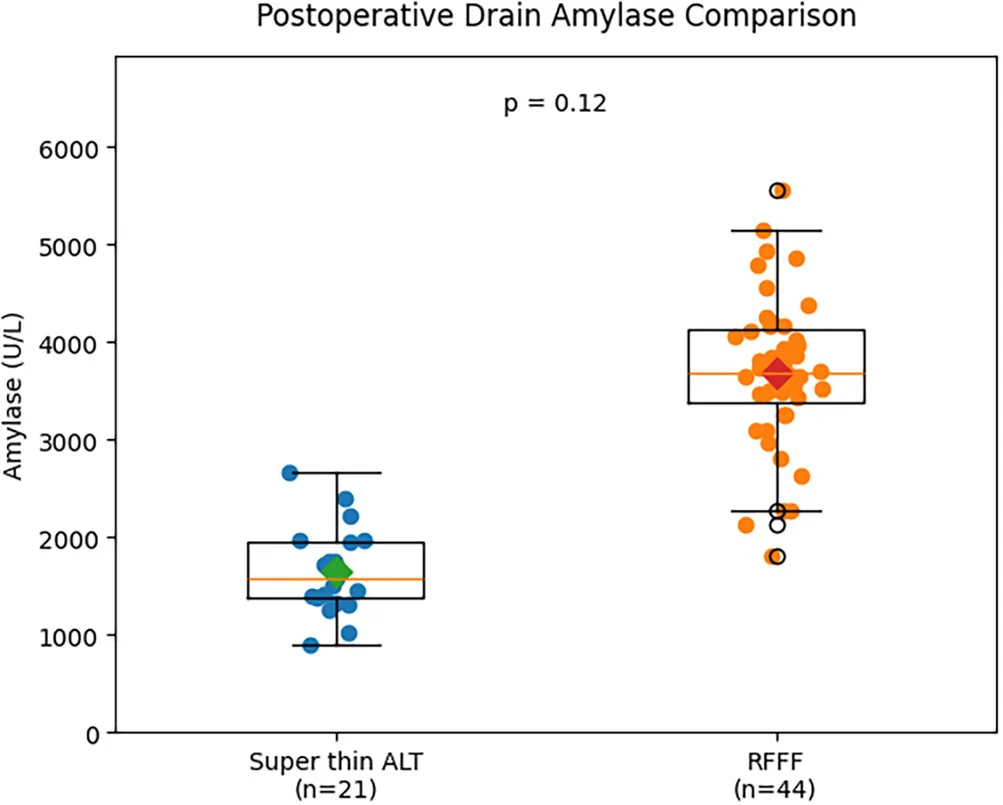
Comparison of postoperative drain amylase levels between super thin ALT flaps and radial forearm free flaps.

Postoperative drain amylase levels, used as a surrogate marker for salivary leakage, are summarized in Table 3. The super thin ALT group demonstrated lower peak amylase levels compared to the RFFF group, suggesting improved conformity and sealing of the reconstructed defect, although no statistically significant differences were observed. Postoperative drain amylase levels are illustrated in Figure 1.

**TABLE 3:** Comparison of postoperative drain amylase levels between super thin ALT flap and radial forearm free flap.

Flap	*n*	Fistula (%)	Peak amylase	POD3	POD5
Super thin ALT	21	3.5	1743	1384	1782
RFFF	44	6.8	3761	1279	2611

Flap design parameters and recipient vessel characteristics of the super thin ALT flaps are presented in Table 4. All reconstructions were performed using end‐to‐end arterial anastomosis and a single venous anastomosis, most commonly to the internal jugular vein. The mean pedicle length was 9.3 cm (range, 6–15 cm), indicating that adequate vascular length can be achieved even with super thin flap elevation.

**TABLE 4:** Flap and recipient vessel characteristics in super thin ALT flap reconstruction.

Parameter	Mean (range) or *n* (%)
Flap width (mean)	7.6 ± 1.2 cm
Flap length (mean)	11.4 ± 1.6 cm
Flap thickness (mean)	8.8 ± 0.9 mm
Pedicle length (mean)	9.3 ± 2.6 cm
Recipient artery	Facial 10 (35.7%)
	Superior thyroid 8 (28.5%)
	Lingual 5 (17.8%)
	Others 5 (17.8%)
Recipient vein	Internal jugular 17 (60.7%)
	Facial 6 (21.4%)
	External jugular 5 (17.8%)

Donor‐site closure methods and associated complications are summarized in Table 5. Most donor sites were closed primarily, with minimal complications such as seroma or delayed wound healing.

**TABLE 5:** Donor site closure methods and complications.

Closure method	Number of cases	Complications (seroma/delayed healing/none)
Primary closure	20	1/0/19
Skin graft	8	0/1/7

**FIGURE 2:**
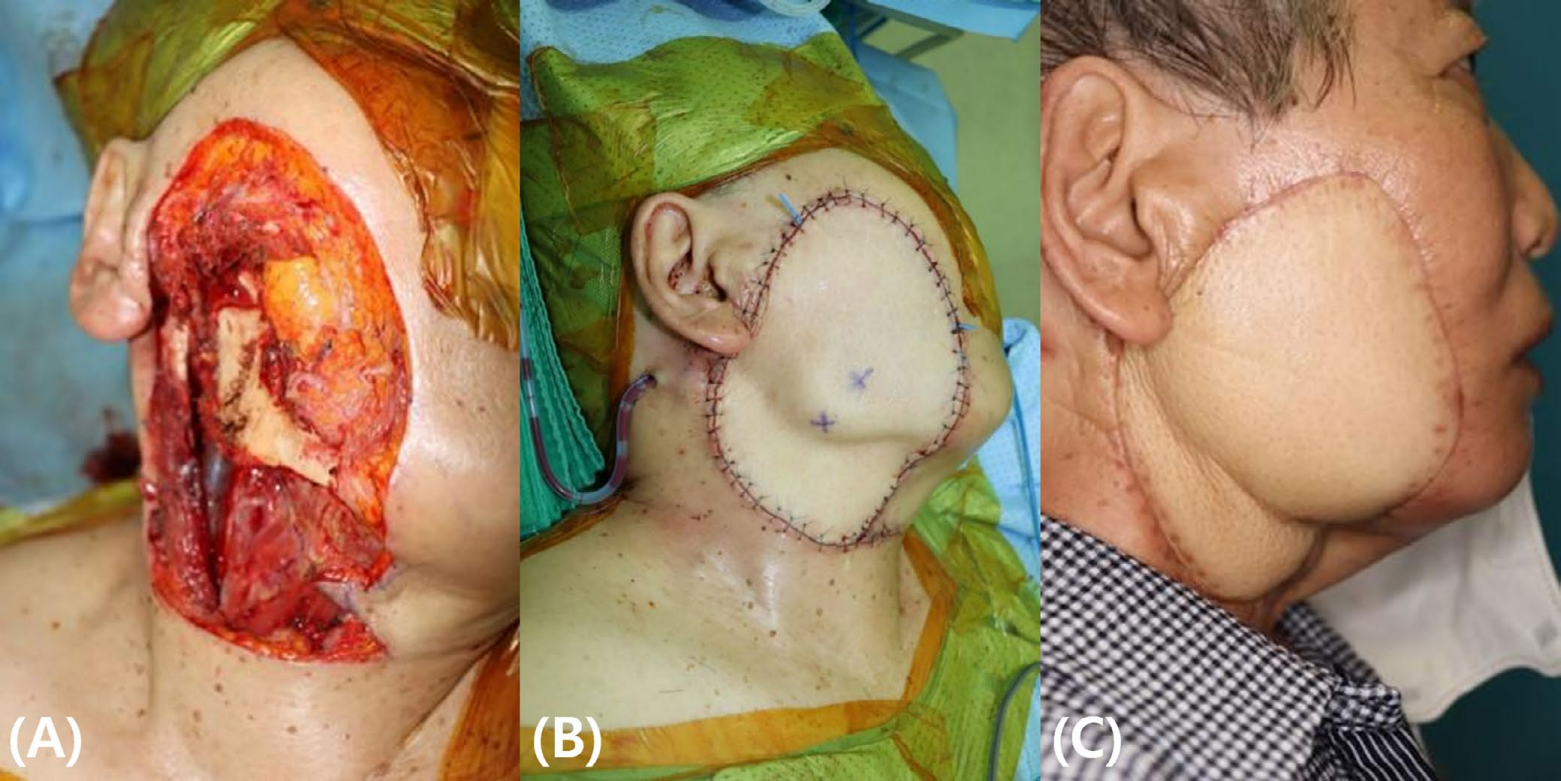
Functional restoration and aesthetic outcome using a super thin ALT flap. (A) Preoperative view showing the skin defect after wide excision of squamous cell carcinoma in the submandibular region. (B) Inset and closure after microvascular anastomosis. (C) Three‐month outpatient follow‐up demonstrating healing and contour.

**FIGURE 3:**
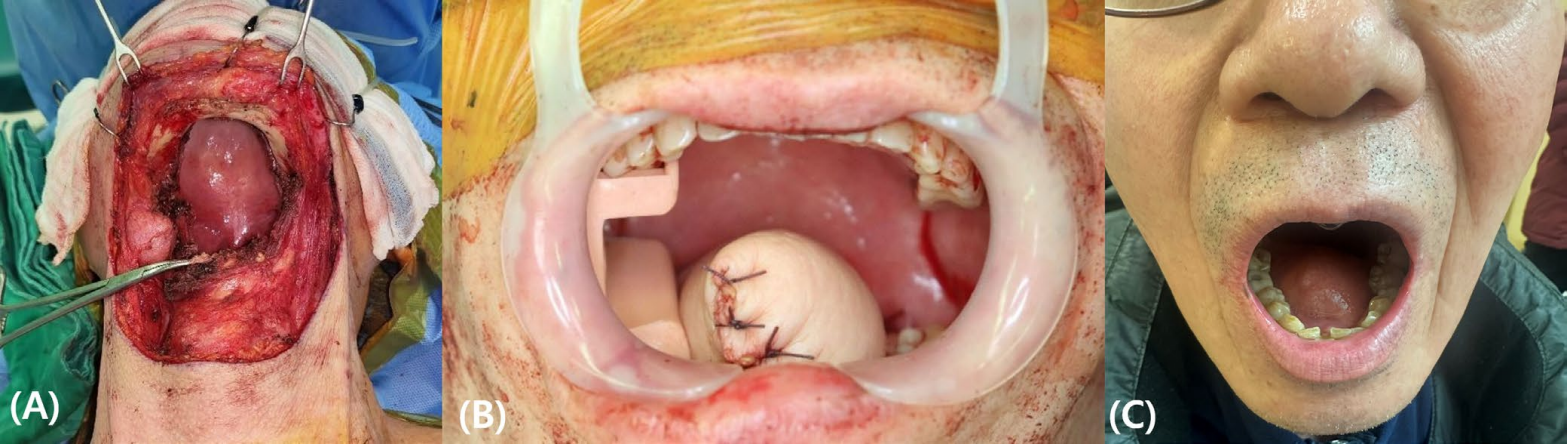
Reconstruction after total glossectomy using a super thin ALT Flap. Intraoperative view immediately after total glossectomy due to tongue cancer. Postoperative view showing flap inset and contour restoration. Two‐year outpatient follow‐up demonstrating stable coverage and functional reconstruction.

**FIGURE 4:**
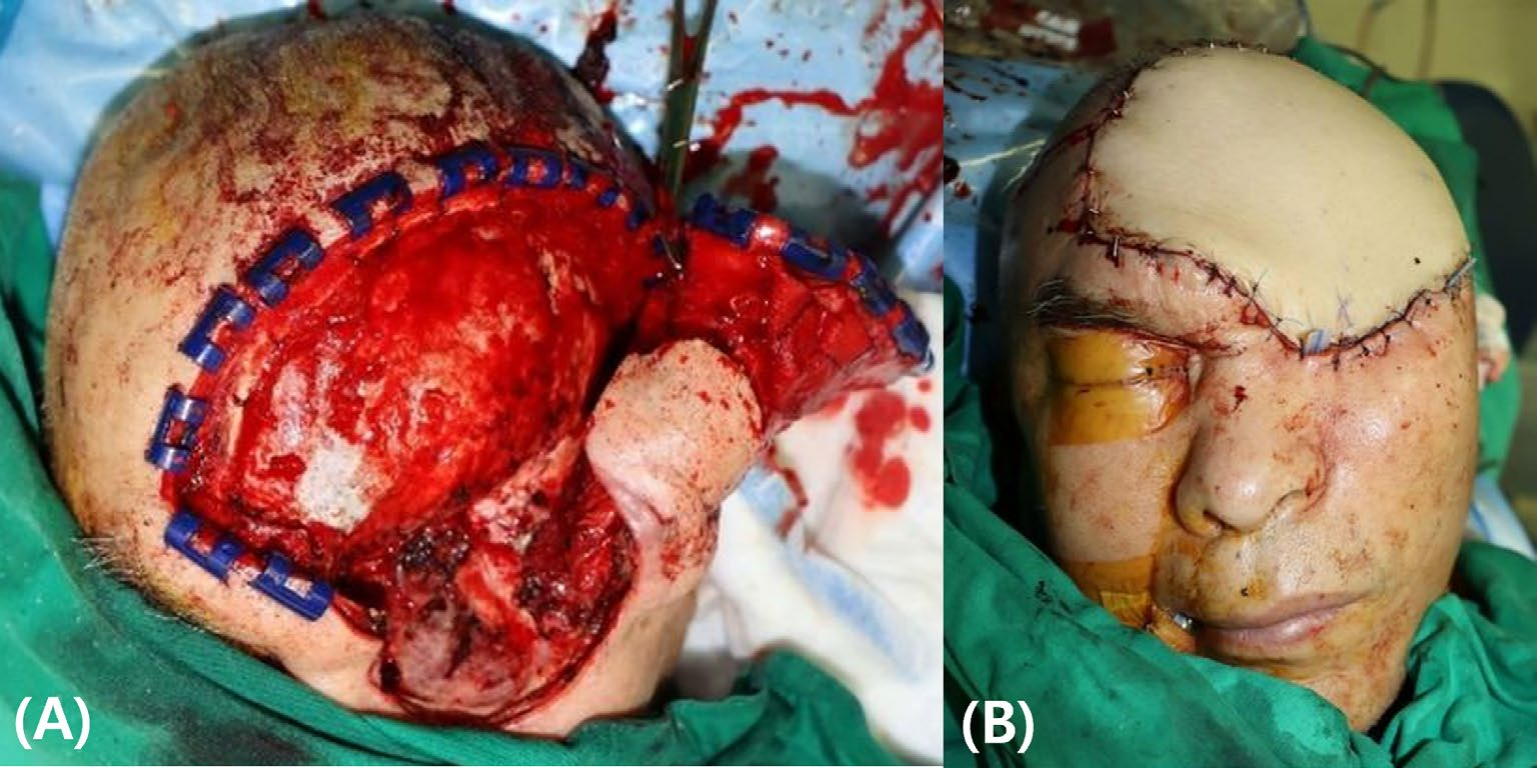
Reconstruction after wide excision in the face using a super thin ALT flap. (A) Immediate postoperative view after wide excision of basal cell carcinoma involving the eyebrow. (B) Intraoperative view showing inset of the super thin ALT flap for defect coverage.

Representative intraoperative and postoperative images are shown in Figures 2, 3, 4, demonstrating defect reconstruction, flap inset, and favorable contour outcomes at follow‐up.

## 4 Discussion

The present study is significant in that it provides a direct comparative analysis between super thin ALT flaps and radial forearm free flaps in head and neck reconstruction, focusing on clinically relevant outcomes such as fistula formation and salivary leakage.

In particular, this study introduces a clear definition of the super thin ALT flap (≤ 10 mm) and demonstrates its safety and reliability when elevated within the superficial fascial layer.

This study demonstrates that super thin ALT flaps can be safely elevated within the superficial fascial layer without compromising vascular reliability. The 100% flap survival rate and absence of partial necrosis confirm that meticulous suprafascial dissection preserves the subdermal plexus and ensures adequate perfusion. These findings are consistent with previous reports demonstrating the safety of flap thinning when performed with appropriate technique (Saint‐Cyr et al. 2009).

Importantly, in this study, super thin ALT flaps demonstrated clinical outcomes comparable to those of radial forearm free flaps (RFFF) in head and neck reconstruction. There were no statistically significant differences in overall complication rates, fistula formation, reoperation rates, or operative time between the two groups. These findings suggest that super thin ALT flaps can achieve similar levels of reliability to RFFF, which has traditionally been considered the standard fasciocutaneous flap for thin and pliable reconstruction (Wei and Mardini 2009; Lakhiani et al. 2012; Wong and Wei 2010).

The higher proportion of prior radiotherapy in the super thin ALT group likely reflects differences in clinical indication and referral patterns between reconstructive options. In our institution, super thin ALT flaps were more frequently selected for secondary or salvage reconstruction following prior oncologic treatments, including radiotherapy and chemotherapy, whereas RFFF was more commonly used in primary reconstruction settings. This difference in patient selection may have influenced postoperative outcomes and should be considered when interpreting the comparative results.

One of the key advantages of super thin ALT flaps lies in their enhanced pliability and improved conformity to complex three‐dimensional defects. In head and neck reconstruction, particularly in intraoral and periorbital regions, incomplete adaptation of the flap can result in microgaps and dead space, which may contribute to salivary leakage and fistula formation. The reduced thickness of the super thin ALT flap allows more precise inset and better surface contact with surrounding tissues, potentially improving functional outcomes (Soutar et al. 1983).

The super thin ALT group demonstrated lower peak amylase levels compared to the RFFF group, suggesting improved conformity and sealing of the reconstructed defect, although the difference was not statistically significant. These findings suggest that improved flap conformity may contribute to enhanced sealing of the reconstructed defect. Additionally, supplementary analysis of cases performed by other departments showed that conventional (bulkier) ALT flaps were associated with higher amylase levels and a greater tendency toward fistula formation, further supporting the importance of flap thinness and pliability in minimizing salivary leakage.

Another advantage of the super thin ALT flap is its versatility in balancing thinness and volume. While thin flaps are advantageous in contour‐sensitive areas, certain defects—such as those involving the tongue—require adequate bulk for functional restoration. In such cases, volume can be effectively achieved through flap folding or partial de‐epithelialization, allowing the surgeon to tailor the reconstruction according to defect requirements. This adaptability broadens the indications of the super thin ALT flap across a wide range of head and neck defects.

Consistent with previous reports, the mean pedicle length in our series (9.3 cm) was sufficient for tension‐free microvascular anastomosis, even with aggressive thinning. The perforator‐centralizing technique described by Suh et al. was applied in all cases, which may have contributed to the excellent vascular outcomes observed in this study (Suh et al. 2021). These findings further support the technical feasibility and reproducibility of super thin ALT flap elevation.

Despite these promising results, several limitations should be acknowledged. This study was retrospective in nature and conducted at a single center with a relatively small sample size. Although a comparative analysis with RFFF was performed, potential selection bias cannot be excluded. In addition, functional and aesthetic outcomes were not assessed using validated scoring systems, and drain amylase levels were not available in all cases. Future prospective, multi‐institutional studies with standardized functional outcome measures are warranted to further validate these findings.

The super thin ALT flap represents a valuable reconstructive option in head and neck surgery (Soutar et al. 1983; Richardson et al. 1997; Fritz and Lin 2014). It maintains the vascular reliability of conventional ALT flaps while providing outcomes comparable to RFFF, with the added advantages of improved pliability, adaptability, and reconstructive precision. These characteristics make it particularly suitable for functionally and aesthetically demanding regions.

## 5 Conclusion

Super thin ALT flaps provide clinical outcomes comparable to radial forearm free flaps in head and neck reconstruction. They represent a safe and reliable option, particularly in contour‐sensitive regions requiring thin and pliable tissue.

## Author Contributions

Conceptualization: Young Chul Suh. Data curation and formal analysis: Kyung Jun Heo. Investigation and methodology: Kyung Jun Heo. Supervision and validation: Young Chul Suh. Writing – original draft preparation: Kyung Jun Heo. Writing – review and editing: Woo Yeol Baek, Jong Won Hong, Won Jai Lee, and Young Chul Suh. All authors read and approved the final manuscript.

## Funding

The authors have nothing to report.

## Disclosure

The authors have nothing to report.

## Ethics Statement

This study was approved by the Institutional Review Board of Severance Hospital, Yonsei University Health System (IRB No. 2025‐1489‐001).

## Consent

Written informed consent was obtained from all patients for publication of clinical images.

## Conflicts of Interest

The authors declare no conflicts of interest.

## Data Availability

The data that support the findings of this study are available from the corresponding author upon reasonable request.
